# Phytotoxicity of *Quillaja lancifolia* Leaf Saponins and Their Bioherbicide Potential

**DOI:** 10.3390/plants12030663

**Published:** 2023-02-02

**Authors:** Maria E. M. Marques, Ana C. de Carvalho, Anna C. A. Yendo, Yve V. S. Magedans, Eliane Zachert, Arthur G. Fett-Neto

**Affiliations:** 1 Plant Physiology Laboratory, Center for Biotechnology and Department of Botany, Federal University of Rio Grande do Sul, Porto Alegre 91501-970, RS, Brazil; 2 Arborea Biotechnology, Center for Biotechnology Start Up Incubator (IECBiot), UFRGS, Porto Alegre 91501-970, RS, Brazil

**Keywords:** soap tree, triterpene saponin, secondary metabolism, germination, seedling

## Abstract

Weeds are major threats to the integrity of agricultural and natural environments due to their invasive and competing potential. Bioherbicides are substances based on natural compounds that are biodegradable and often have low residual effects. Plant species able to produce and release phytotoxic compounds may represent effective bioherbicide sources. Leaves of *Quillaja lancifolia* D.Don (formerly *Q. brasiliensis* (A.St.-Hil. & Tul.) Mart.) produce water-soluble specialized metabolites of the saponin class that could be evaluated for phytotoxic activity and potential as natural herbicides. This study was conducted to examine the impacts of *Q. lancifolia* total saponins aqueous extract (AE) at 4 and 10% (*w*/*v*) and of two combined reverse-phase chromatography purified saponin fractions (QB) at 1 and 2% (*w*/*v*) on morpho-physiological parameters of *Lactuca sativa* (lettuce) and *Echinochloa crus-galli* (barnyardgrass) in pre- and post-emergence bioassays. QB was only tested in pre-emergence assays. In pre-emergence bioassays, the germination rate and germination kinetics were determined. Post-emergence evaluations included effects on seedling morphology, root and shoot length, dry mass, and chlorophyll content. Osmotic potential and pH analyses ruled out roles for these factors in the observed responses. AE had a high inhibitory impact on the germination of both lettuce and barnyardgrass. QB at 1% and 2% (*w*/*v*) significantly decreased the growth of lettuce seedlings germinated in its presence by more than 10-fold. Phytotoxic effects on the post-emergence growth of lettuce, especially at the highest concentration tested of AE (10% *w*/*v*), was also observed. The presence of quillaic acid-based triterpene saponins in AE and QB was confirmed using different analytical methods. Therefore, both saponin-enriched fraction and aqueous extracts of *Q. lancifolia* inhibited tested plant growth and development. The water solubility of saponins and the availability of a sustainable source of these molecules from the leaves of cultivated young *Q. lancifolia* plants make them attractive candidates for use as bioherbicides.

## 1. Introduction

The combination of efficient agricultural land use with biodiversity conservation is a challenge [1]. It is estimated that agriculture will need to provide almost 50% more food by 2050 [2]. To sustain this level of production and knowing that 20–40% of global crop yields are lost each year to pests and diseases, pesticide use in agriculture has increased in recent years. Due to its extensive planting area, Brazil is one of the three largest consumers of pesticides in the world [2,3].

“Pests” are considered invasive organisms or pathogens of crops or forest species [4] and include animal pests (insects, mites, nematodes, rodents, slugs, snails, birds), plant pathogens (viruses, bacteria, fungi, chromists), and weeds (i.e., competitive plants) [5]. It has been estimated that weeds represent the most damaging category of pests in agriculture [5,6]. The Weed Science Society of America defines a weed as a plant that causes economic losses or ecological damage, creates health problems for humans or animals, or is undesirable where it is growing [7]. Weeds negatively affect crop yields due to a number of features, including: (a) capacity to grow in disturbed habitats, (b) high environmental plasticity, (c) ability to physically hinder or smother crop growth, (d) capacity to compete with crops for light, nutrients, moisture, and space, (e) rapid emergence, seedling growth, and quick maturation, (f) production of a large numbers of seeds per plant, (g) ability to release natural substances that inhibit crop growth (allelopathy), and (h) host pests or pathogens that may contaminate crops [8,9].

Although pesticides prevent, destroy, and repel most pests growing on crop plants, their continued use can have a significant impact on the environment and human health by contaminating water bodies, air, soil, and food. Moreover, unintentional negative effects on local biodiversity are often observed [10,11]. Thus, there is an increasing demand for pesticides that are safe for the environment and may replace or complement synthetic management methods [12]. This is an important concern in organic farming, given its expansion and the need for environmentally safe management tools [9,10]. Weed management techniques can vary (manual, mechanical, chemical, and biological control) but depend mostly on the use of synthetic herbicides (pre-emergent and post-emergent) [11,13]. Hence, it is important to search for new and effective alternatives for weed management.

Substances obtained from living organisms, e.g., the natural metabolites produced during growth and development, can be ingredients of bioherbicides. Although the use of these metabolites can be efficient and beneficial, few conventional herbicides are derived from natural compounds [14,15]. Accordingly, the use of plant species able to produce and release phytotoxic compounds may represent an effective tool for use alone or in association with other plant protection methods for weed management [12,16].

Saponins are specialized metabolites with complex chemical structures and high variability that are involved in the environmental adaptation of plants [17,18]. These molecules are widely distributed among plants and have several significant industrial and pharmacological applications [19]. Some studies have reported phytotoxic or allelopathic properties for saponins [20,21,22,23,24,25,26], which suggests that untested saponins could be evaluated as potential bioherbicides [14].

*Quillaja lancifolia* D.Don (formerly *Q. brasiliensis* (A.St.-Hil. & Tul.) Mart.) (Quillajaceae), known as the soap tree, is distributed throughout Southern Brazil [27]. The species is well known for the abundant presence of saponins and, therefore, a large spectrum of biological activities, including immunoadjuvant properties [28,29], antifungal, and antiherbivore effects [30]. The structure of *Q. lancifolia* saponins is remarkably similar to that of *Quillaja saponaria* Molina bark saponins [31]. The latter is a related Chilean species that has been widely used as an adjuvant in vaccine formulations and is one of the main sources of industrial saponins present in plants. The commercially used saponin fraction obtained from barks of *Q. saponaria* is known as Quil-A^®^ [19]. Studies have reported that *Q. saponaria* shows strong aphicidal, deterrent, nematocidal, molluscicidal, and antifungal activity, indicating that the plant could potentially be used as a biopesticide [32,33,34,35,36]. Some of these features have also been observed for leaf saponins of *Q. lancifolia* [30]. If *Q. lancifolia* saponins prove to be phytotoxic, there is also potential use of these natural products as bioherbicides. In addition, saponins may be used in combination with other molecules at lower concentrations, thereby preventing resistance development in plant enemies. Regardless of its potential use as a biopesticide or bioherbicide, *Q. lancifolia* leaf saponins are a more sustainable and easily renewable alternative compared to *Q. saponaria* bark saponins since they do not require phloem stripping or the use of adult native forests [37]. Miniclonal gardens of young *Q. lancifolia* provide a readily available year-round source of leaf biomass [19].

Herein, the phytotoxic activities of leaf aqueous extract and purified saponin fractions of *Q. lancifolia* on different morpho-physiological parameters of lettuce and barnyard grass were examined. Parameters included germination, growth, chlorophyll concentration, and the presence of morphological anomalies. Exposure of soil-like substrate to *Q. lancifolia* extracts, followed by washing with water, was used to assess retention on the substrate and potential residual effects of bioactive metabolites. Chemical analyses of aqueous extracts and purified saponin fractions were also carried out.

## 2. Results

### 2.1. Phytotoxicity Assay

#### 2.1.1. Osmotic Potential, pH of Extracts, and Presence of Saponins

PEG solutions up to 0.02 M did not significantly affect the emergence of lettuce and barnyardgrass seeds. Germination reached between 80 and 100% in both cases, being statistically equivalent to respective water controls. Since the osmotic potential of the 0.02 M solution was equivalent to that of the 10% *Q. lancifolia* extract, it was determined that, up to this concentration, the effects on seeds were not due to any osmotic limitation (Table S1). Similarly, the pH of the extract was in the 4–6 range, which was not capable of inhibiting germination of the test species.

Triterpene saponins’ presence in aqueous extracts and purified fraction QB-90 were confirmed unequivocally by the analytical tests carried out. The TLC data indicated the presence of saponins matching the Rf of Quil-A, a commercial fraction obtained from *Q. saponaria* bark. Although, as expected, other compounds (e.g., phenolics) were present in aqueous extracts, a prominent band matching Quil-A was evident (Figure S1). Further analyses by HPLC revealed peaks with saponin spectral features within the elution times expected for these metabolites (Figure S2). GC-MS analysis of QB-90 after acid hydrolysis yielded quillaic acid as the main aglycone (Figure S3), confirming the triterpene saponin core identity.

#### 2.1.2. Pre-Emergence Bioassay

**Figure 1:**
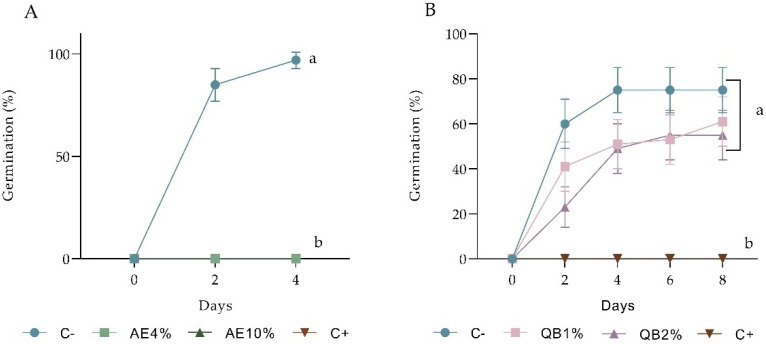
Pre-emergence bioassay with lettuce. Effects of (**A**) aqueous extracts (AE) (at 4 and 10%) and (**B**) saponin fraction (QB) (at 1 and 2%) on the number of germinated seeds of *L. sativa* at the end of 4 days and 8 days, respectively. C- represents the negative control (distilled water), and C+ represents the positive control (NaCl). Bars show 95% confidence intervals (Wilson score interval). Treatments not sharing the same letter at the final sampling time differ in germinability (*p* ≤ 0.05).

**Figure 2:**
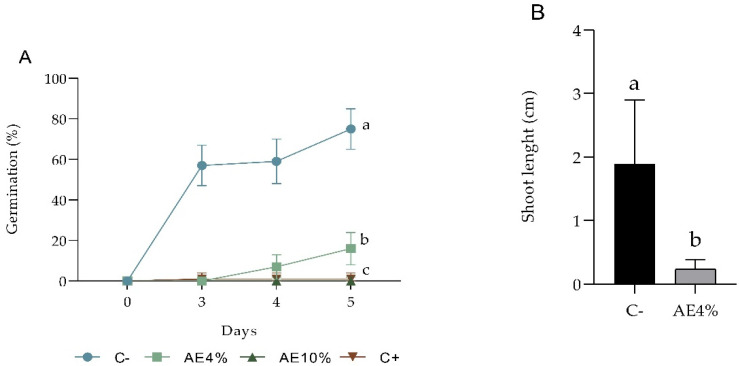
Pre-emergence bioassay in barnyardgrass. (**A**) Seed germination in distilled water (C-), aqueous extract (AE) at 4%, AE at 10% and NaCl (C+) and (**B**) seedling growth (shoot length) at the end of 5 days in treatments that showed germination. In (**A**), bars show 95% confidence intervals (Wilson score interval). Treatments not sharing the same letter at the final sampling time differ in germinability (*p* ≤ 0.05). In (**B**), bars represent the mean ± SD. Treatments are significantly different by *t*-test (*p* ≤ 0.05).

**Figure 3:**
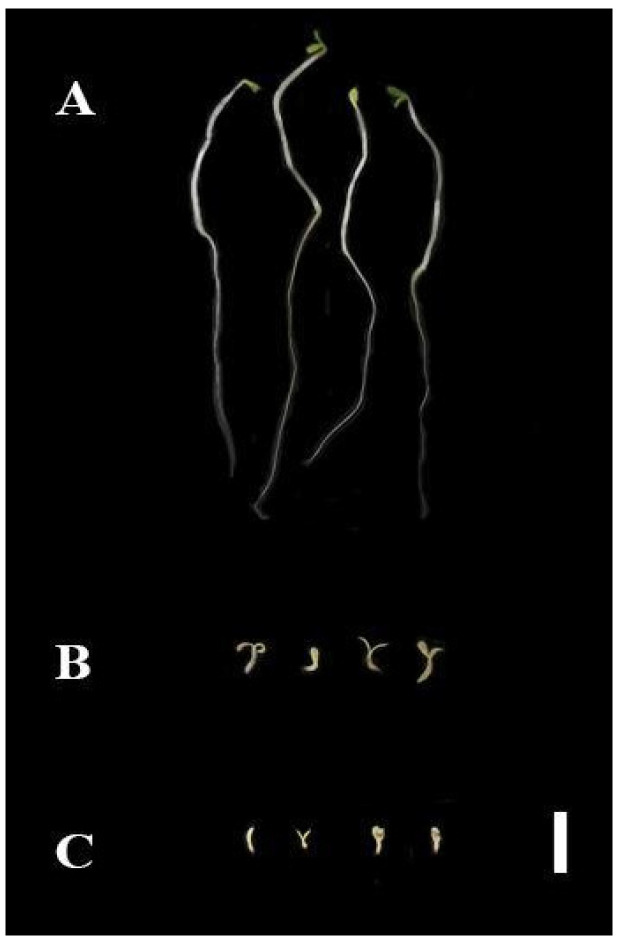
Pre-emergence bioassay with lettuce. Seedings at the end of 8 days of application of (**A**) distilled water, (**B**) purified saponin (QB) at 1%, and (**C**) QB 2%. Bar = 1 cm. Statistical data on whole seedling length (cm): distilled water (7.65 ± 0.93 a); QB at 1% (0.60 ± 0.14 b) and QB at 2% (0.38 ± 0.05 b). Numbers in brackets are means ± SD. Means sharing the same letter do not differ by one-way ANOVA and Tukey test (*p* ≤ 0.05).

Both AE 4% and 10% caused significant inhibitory effects on seed germination. In lettuce, no germination was recorded after 4 days, while the negative control had 97% germinability (Figure 1A). Barnyardgrass had 16% germination in 4% AE and 75% in the negative control; the 10% extract completely inhibited germination (Figure 2A). Both 4 and 10% AE reduced not only the percentage of germination but also the speed of the process in the two species (Table S2). Purified saponins (QB fraction) did not inhibit lettuce germination significantly (Figure 1B). On the other hand, the growth of seedlings germinated in the presence of QB was severely impaired (Figure 3).

#### 2.1.3. Post-Emergence Bioassay

**Figure 4:**
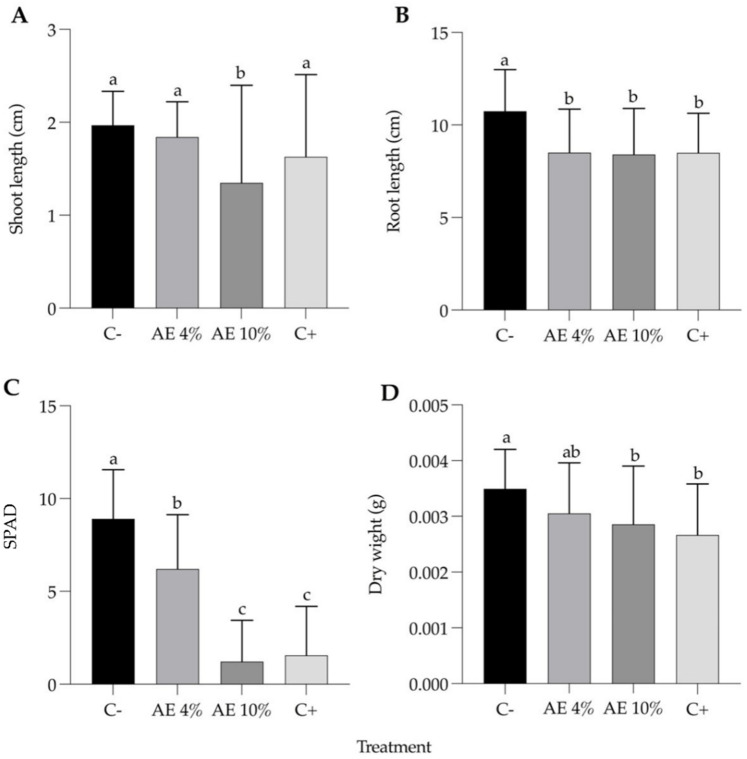
Post-emergence bioassay with lettuce. Parameters (7 days after treatment): (**A**) shoot length; (**B**) root length; (**C**) chlorophyll content (SPAD readings); and (**D**) seedling dry weight. Abbreviations: C- (distilled water), AE 4% (aqueous extract at 4%), AE 10% (aqueous extract at 10%), and C+ (NaCl). Bars represent the mean + SD. Different letters indicate significant differences by Kruskal–Wallis test and Dunn’s test (*p* ≤ 0.05).

**Figure 5:**
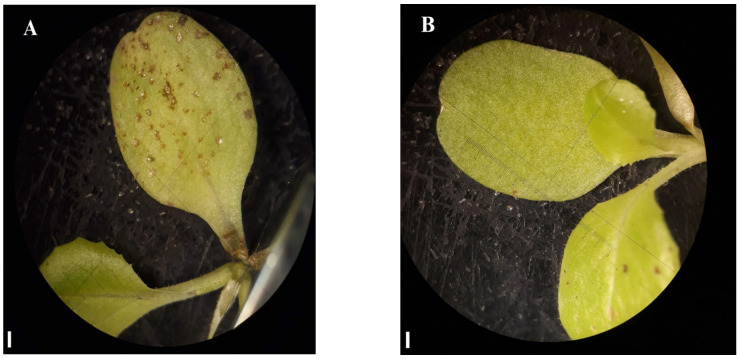
*Lactuca sativa* leaves at day 7 in the post-emergence bioassay treated with (**A**) aqueous extract (AE) at 4% and (**B**) negative control (distilled water). Note the brown spots on the leaves of (**A**). Bar = 1 mm.

Seven days after the application on lettuce seedlings, exposure to both 4 and 10% AE yielded significant differences from the negative control in every parameter analyzed, except for shoot length and dry weight in the lower concentration (4%) (Figure 4). The chlorophyll content of AE 10% seedlings decreased as much as that of their positive control treatment (NaCl) counterparts (Figure 4C). Plants in AE 4% showed abnormalities and brown spots on the leaves (Figure 5). Moreover, most of the seedlings of the AE 4% were notably fragile. The loss of seedling viability in AE 10% (24%) was comparable to the one recorded in the positive control (16%) (Table S3).

In barnyardgrass, no significant difference was observed in root length, shoot length, chlorophyll content, and seedling dry weight between the treatments and the controls (Figure S4). However, the plants from the 10% AE appeared wilty (Figure S5).

In the additional post-emergence bioassay growing lettuce in Petri dishes, a significant reduction was observed in root and shoot elongation of seedlings in both AE treatments (4% and 10%) and the positive control (NaCl) (Figure S6). No significant difference was observed in the seedling dry mass (Figure S6C). The chlorophyll content was not measured in this experiment because the plants were too small.

#### 2.1.4. Substrate Leaching Bioassay

**Figure 6:**
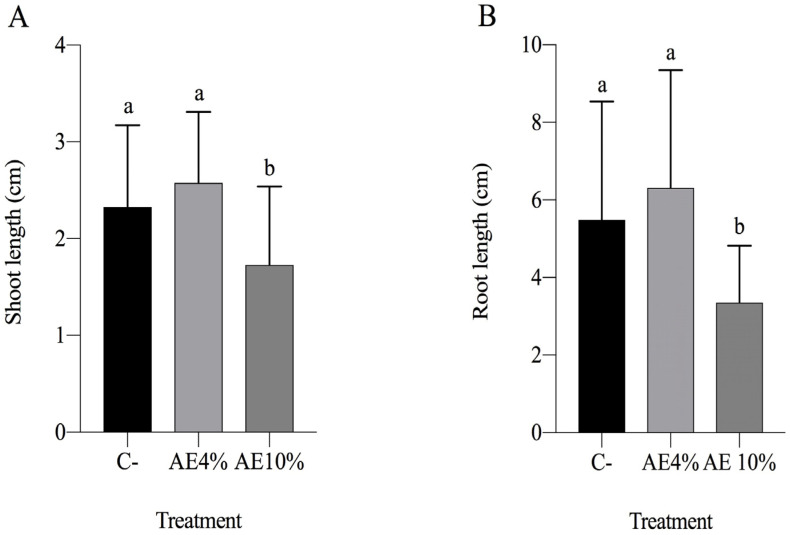

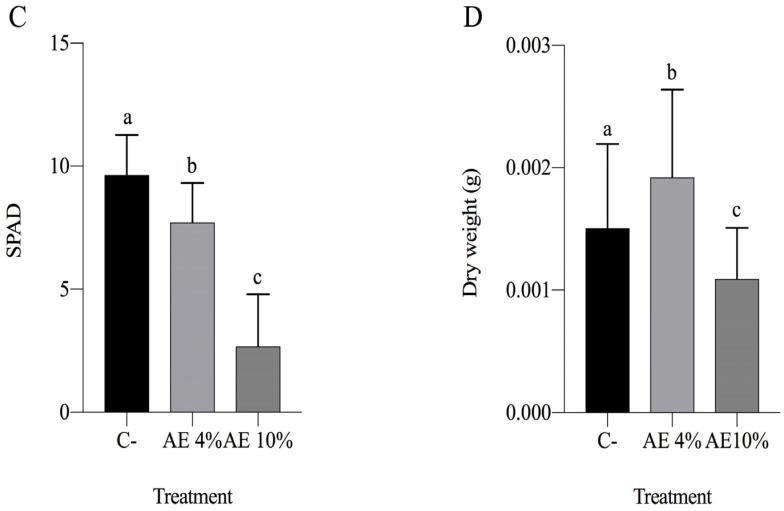
Substrate leaching bioassay with lettuce. Parameters (12 days of application of treatments): (**A**) shoot length, (**B**) root length, (**C**) chlorophyll content, and (**D**) seedling dry weight. Abbreviations: C- (distilled water), AE 4% (aqueous extract at 4%), AE 10% (aqueous extract at 10%). Bars represent the mean + SD. Different letters indicate a significant difference by one-way ANOVA and Tukey test (*p* ≤ 0.05).

The soil-like substrate leaching bioassay carried out with lettuce evaluated germination rates and plant development. Regarding seedling growth, the 10% extract resulted in a significant reduction of shoot length, root length (Figure S7 and Figure 6A,B) and dry mass (Figure 6D). In addition, plants treated with the extracts had a dose–response decrease in chlorophyll content (Figure 6C). Interestingly, the germination rate was higher with AE 10% and AE 4% (56% and 51%, respectively), while the negative control had only 28% seedling emergence (Figure S8). The dry weight was also higher in the 4% extract compared to the control (Figure 6D).

**Figure 7:**
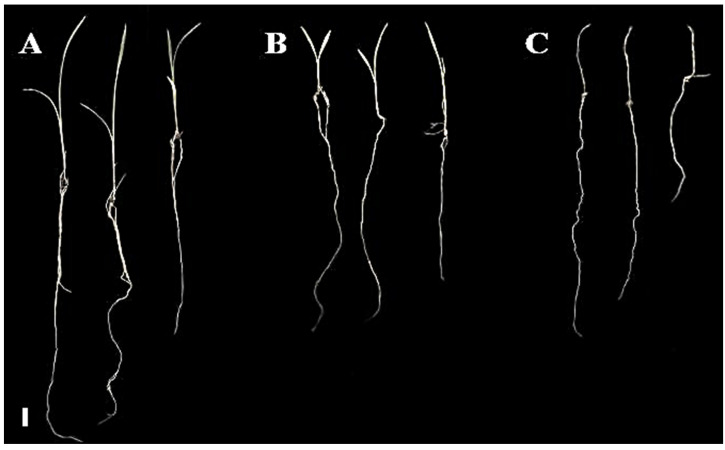
Seedlings of barnyardgrass at the end of 8 days in the leaching bioassay. (**A**) Negative control (plants treated with distilled water); (**B**) plants with aqueous extract (AE) at 4% and (**C**) plants with AE at 10%. Bar = 1 cm.

**Figure 8:**
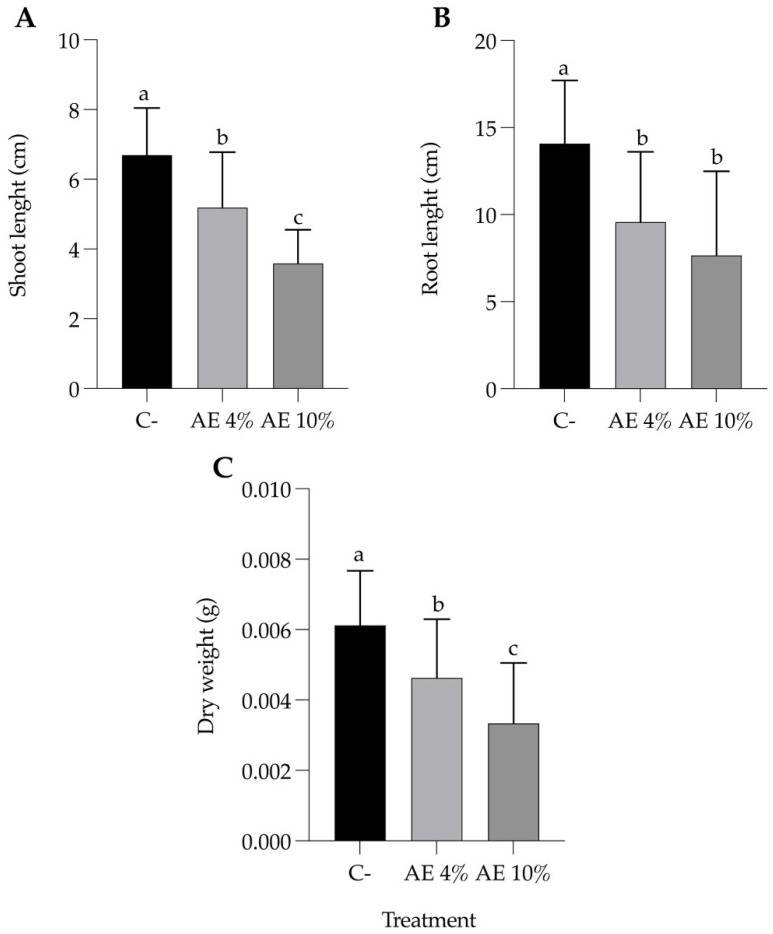
Substrate leaching bioassay with barnyardgrass. Parameters after 8 days of leaching (and 4 water applications): (**A**) shoot length, (**B**) root length, and (**C**) seedling dry weight. Abbreviations: distilled water (C-), aqueous extract at 4% (AE 4%), and aqueous extract at 10% (AE 10%). Bars represent the mean + SD. Different letters indicate significant differences by one-way ANOVA and Tukey test (*p* ≤ 0.05).

Barnyardgrass plants under extract treatments showed significant growth inhibition, in contrast with those of the negative control (Figure 7). AE 4% and AE 10% revealed a concentration-dependent inhibitory effect on seedlings in terms of shoot length and dry weight (Figure 8A,C).

## 3. Discussion

Possible significant impacts of nonspecific factors on the phytotoxic activities of *Q. lancifolia* aqueous extracts and purified saponins at the concentrations used were ruled out. Therefore, the validity of the subsequently recorded data was confirmed. Osmotic and pH values were within non-inhibitory ranges for both tested species [38,39,40].

Pre-emergence bioassays using 4% and 10% aqueous extracts of *Q. lancifolia* against both lettuce and barnyardgrass revealed a significant reduction of germination rates and root and shoot length of the emerged seedlings. In *L. sativa,* the extracts completely inhibited germination. Extracts and QB fraction significantly inhibited one or more parameters, such as germination percentage and shoot and root length. Considering that even at low concentrations of AE, germination was suppressed, it appears that the extracts showed phytotoxic potential that could be at least partly attributed to the saponins present in the plant. Indeed, analytical methods consistently showed the presence of triterpene saponins with typical quillaic acid aglycone [19,31]. The possible participation of saponins in the phytotoxic effects is also supported by the fact that seeds germinated in the presence of the purified saponin fraction QB-yielded seedlings severely reduced in size. Further studies of these compounds could lead to possible use as an effective pre-emergence bioherbicide.

Phytotoxic effects on the post-emergence growth of lettuce were also observed, especially at AE 10%. Under the AE 4% treatment, numerous leaves exhibited abnormalities that could explain the significantly reduced chlorophyll content recorded. Although the effects on post-emergence were not as intense as those in the germination experiments, *Q. lancifolia* extracts also had phytotoxic effects on seedling growth. In the leaching bioassay, however, there was a significant decrease in the inhibitory effect of the extracts on lettuce, particularly at 4% AE. This could lead to possible use as a post-emergence bioherbicide, alone or in combination, effective at pre- and post-germination with a reduced residual effect.

However, the lack of significant changes shown in the post-emergence bioassays with barnyardgrass can indicate that the extracts are not as effective in repressing the seedling growth of *E. crus-galli* as they are inhibiting its germination. This possibly reflects a higher resistance to biotic stresses in weeds [8]. On the other hand, the leaching bioassay with barnyardgrass showed a significant reduction in the root and shoot length as well as dry weight of plants treated with AE. Perhaps, for this species, longer exposure to the extract residue could affect plants negatively.

Ideally, a bioherbicide residue on the soil surface should be able to undergo leaching and/or degradation, thus avoiding the exposure of subsequent crops to potentially phytotoxic compounds [11]. The leaching assay with lettuce is also considered a pre-emergence experiment since it used seeds grown on substrates exposed to the extracts. Since mixtures of sand and vermiculite are only soil-like substrates, the data obtained with them cannot be directly extrapolated to natural soil conditions. Nonetheless, the use of these substrates provides useful information on the overall behavior of phytotoxic agents in soil. The higher dry biomass of seedlings in AE 4% relative to the water control may indicate a nutritional effect of the extracts caused, for example, by the presence of sugars in saponins [41]. The application of the 10% extract, nonetheless, resulted in lower growth and chlorophyll content, perhaps due to a negative impact on photosynthesis [42].

Antiherbivore and antifungal effects of *Q. lancifolia* saponins were observed in the 1 to 2% concentration range [30]. For saponins to constitute a potential biopesticide, the absence or limited phytotoxic effects at concentrations in which they can inhibit herbivores and pathogens are necessary. Further studies focusing on the compatible use of these molecules in biopesticide and bioherbicide formulations are needed.

In most cases, as has been reported for other saponins, the activity of these triterpenes depends essentially on their effects on membranes [43]. As a result of their amphipathic nature, saponins can interfere with the membrane structure, affecting its integrity and functionality [44]. It seems likely that the growth inhibitory effects recorded in test plants are at least partly caused by saponins present in extracts and their fractions on cell and organelle membranes. However, other compounds, as well as synergistic interactions among metabolites, can also be involved, particularly in AE treatments.

Taken together, the data suggest that AE and QB of *Q. lancifolia* may be considered for use in organic agriculture both as pre- and post-emergence bioherbicide components. In a preliminary assessment, it seems that these agents may be effective for broad leaf and grass weeds. Given the surfactant properties of saponins and based on pilot tests, their application using ordinary sprayers is not difficult. Although it is not possible to estimate more precisely at this point, doses per hectare and the costs of treatment are not likely to be prohibitive. Support for this possibility is based on the renewable and continuous source of saponin supply from the leaves of cultivated plants, as well as on the relatively low active concentrations required. In addition, the combined use of these agents as adjuvants with other products may further contribute to economic feasibility.

## 4. Conclusions

In summary, *Quillaja lancifolia* aqueous extracts and purified saponin fractions showed strong phytotoxic activity against *E. crus-galli* and *L. sativa* (with greater injury levels on the latter) and weed-suppressing abilities. These findings warrant further studies to evaluate the possible uses of these agents in formulations of bioherbicides. Key features of *Q. lancifolia* saponins, such as their water solubility and sustainable supply from leaves obtained in cultivated clonal gardens, are important for potential deployment as bioherbicides. Future work is required to better understand the mechanisms of action of these phytotoxins, notably their interaction with biological membranes and their components.

## 5. Materials and Methods

### 5.1. Plant Material and Extracts

#### 5.1.1. Plant Source and Extract Preparation

Approximately 5 kg of fresh *Q. lancifolia* leaves were collected from adult plants (about 10 m tall of unknown age) growing in the rural zone of the city of Canguçu, RS, Brazil (31°23′42″ S–52°40′32″ W) in the early fall of 2018 (28 March 2018) (harvest authorization by the Brazilian Board of Management of Genetic Resources—CGEN under number 010540/2011-3). Harvest was done on a sunny day with temperature variations between 17 and 24 °C. Monthly precipitation was typical for the area, around 120 mm. The climate of the site of collection is warm temperate Cfb (Köppen classification).

A voucher specimen was deposited at the ICN Herbarium of the Federal University of Rio Grande do Sul (142,953). Air-dried powdered leaves were extracted in distilled water (1:10, *w*/*v*) for 8 h, filtered, and partitioned with ethyl acetate, followed by lyophilization of the aqueous phase, yielding aqueous extract (AE). AE was submitted to reverse-phase chromatography and gradient of water and methanol to obtain fraction QB-90, as previously described [28]. QB-80 was obtained using the same protocol. QB-80 and QB-90 were also analyzed by thin-layer chromatography (TLC) to further confirm fraction isolation. The two purified saponin fractions were combined for the bioassays constituting QB.

#### 5.1.2. Extract and Saponin Chemical Characterization

Characterization of AE and QB was initially performed by TLC. Silica gel aluminum plates (F254, Supelco, Darmstadt, Germany) were used as the stationary phase. The mobile phase was n-butanol:acetic acid:water (5:1:4, *v*/*v*). Plates were revealed using the anisaldehyde-sulfuric acid protocol [28]. Additional confirmation of saponin presence was obtained by high-performance liquid chromatography (HPLC) analysis using a Shimadzu chromatographer equipped with a PDA-UV detector. Saponins were tracked at 214 nm. The HPLC method was based on Wallace et al. [45] with minor modifications. Finally, Gas Chromatography-Mass Spectrometry (GC-MS) analysis of acid-hydrolyzed saponins was carried out with an oven set to 120 °C for 1 min, then increasing at 6 °C/min to 320 °C. The final hold time was 11.6 min. Spectral data were generated with electron impact ionization at 70 eV (positive mode). Data were acquired in full scans ranging from *m*/*z* 50 to 550. The Agilent MassHunter workstation (version B.07 service pack 2) was used for peak integration of total and extracted ion chromatograms and extraction of mass spectra [46].

### 5.2. Phytotoxicity Assay

#### 5.2.1. Target Species

The experiments on the phytotoxic effect of the extract were carried out with diaspores (herein referred to as seeds) of *Lactuca sativa* L. (a common model in allelopathy studies), as well as seedlings of the same species; and with seeds and seedlings of *Echinochloa crus-galli* L.P.Beauv (an economically relevant weed species, particularly in rice fields). For these assays, commercially available lettuce seeds were used (Isla^®^, batch 116377-001, Porto Alegre, Brazil), and barnyardgrass seeds (batches grown at the agronomy campus of UFRGS and harvested in late summer/early fall) were kindly provided by the Department of Crop Plants—Faculty of Agronomy, UFRGS.

#### 5.2.2. Extract Osmotic Potential and pH Measurements

Both the osmotic potential and pH of the extracts were determined in triplicate. The osmotic potential of *Q. lancifolia* extracts was measured in different concentrations (0.1%, 1%, 2%, 4%, 10%, 20%, 40%) with a refractometer-based curve prepared with sucrose. To determine the influence of the osmotic potential of the extracts on both lettuce and barnyardgrass germination bioassays, additional tests were performed with solutions of polyethylene glycol (PEG-6000) [38] at the following concentrations: 0.01 M, 0.02 M, and 0.03 M. This experiment was carried out using the same germination procedure (i.e., pre-emergence) bioassays (Table S1 and Section 5.2.3). The osmotic potential of the different concentrations of PEG was also measured with a refractometer, and it was possible to correlate the solution of PEG at 0.02 M with the 10% *Q. lancifolia* aqueous extract. Extract pH values were determined with a pH meter.

#### 5.2.3. Pre-Emergence Bioassay

The pre-emergence bioassay analyzed the germination of *L. sativa* (lettuce) and *E. crus-galli* (barnyardgrass). Petri dishes (9 cm in diameter) lined with qualitative filter paper were used, and assays were conducted in a growth chamber (25 °C, 12 h/12 h dark/light, 40 µmol photons·m^−2^·s^−1^).

For each one of the species, the Petri dishes were separated into experimental groups and the control, each containing 25 seeds per plate, with 3 plates per experimental group. Seeds were treated with 5 mL in each Petri dish of the AE of *Q. lancifolia* (at 4 and 10% *w*/*v*), distilled water (negative control), and NaCl 0.5 M (positive control). In the case of lettuce, QB at 1% and 2% (*w*/*v*) were also evaluated.

Germination parameters were measured daily (germination time) or on the 5th day of incubation (germination percentage, root and shoot length). The presence of morphological abnormalities was recorded. Germination velocity indexes (GVI) were calculated [47]:

GVI = N1/D1 + N2/D2 + … + Nn/Dn

where: GVI = germination velocity index; N = number of seeds germinated within the day interval of counting; D = days after sowing until data collection.

An additional experiment using purified saponin fraction QB was performed with lettuce under the same growth conditions described above. Germination and seedling length were analyzed, with treatments consisting of 1 and 2% of QB and controls, in this case ending on the 7th day of incubation.

#### 5.2.4. Bioassay for Post-Emergence

To obtain lettuce seedlings for the effect on initial growth, seeds were put in separate pot trays (each slot with a volume of 15 mL, one plant per slot) containing autoclaved substrate (washed sand: vermiculite—ratio 1:1; *v*/*v*) and kept for 12 days under controlled light and temperature (photoperiod of 16 h per day; 25 ± 2 °C, irradiance 50 µmol·m^−2^·s^−1^). On the 12th day, plants from germinated seeds were exposed to AE (at 4 and 10% *w*/*v*) and controls (2 mL per slot containing a single plant and sprinkling to dew point). Barnyardgrass seeds were kept for 13 days on the same substrate growth conditions described above, and on the 14th day, seedlings were exposed to the treatments (AE at 4 and 10% *w*/*v*).

Seven days after treatment application, growth parameters were measured (radicle length, shoot length, and whole seedling dry mass). Root and shoot length were recorded with a ruler, and oven dry mass at 60 °C was obtained in an analytical balance. In addition, for lettuce, the chlorophyll content was measured with a SPAD chlorophyllometer (Minolta, Osaka, Japan). With barnyardgrass, due to restrictions of leaf size on the SPAD reading probe, a conventional spectrophotometric evaluation of acetone chlorophyll extracts was carried out [17]. Each individual plant was considered an experimental unit. The number of replicates per treatment and per sampling time was 50 for root length, shoot length, whole seedling dry weight, and SPAD readings. For spectrophotometric chlorophyll measurements, a pooled set of 5 plants was the experimental unit and 4 replicates were used per treatment and sampling time.

In another set of experiments, *Q. lancifolia* extracts and control treatments were applied to post-emergent 12-day-old lettuce seedlings grown in Petri dishes. After 12 days in growth chamber conditions as described above, 5 mL of extracts and controls were applied to each dish containing 25 plants and replicated 3 times. Plants were measured after 7 days of application (root length, shoot length, and whole seedling dry mass).

#### 5.2.5. Substrate Leaching Bioassay

To evaluate whether the extract could be leached from a soil-like substrate, two different bioassays were performed. With *L. sativa*, 50 individual slots of pot trays (each slot with a volume of 15 mL) containing autoclaved soil-like substrate (washed sand: vermiculite—ratio 1:1; *v*/*v*) were inoculated with 2 mL of AE at concentrations of 4 and 10%. In addition, the area comprised of 50 slots was superficially sprayed with 30 mL of the respective extract. Then, each slot was subjected to leaching with 10 mL of distilled water every 2 days until completing 8 days (i.e., 4 washings). In the day of the last water application, 2 lettuce seeds were sown per slot to evaluate germination for 12 days. Root length, shoot length, and seedling dry mass were also recorded at the end of this period. The experiment with barnyardgrass evaluated the leaching potential of seedlings grown on the same untreated substrate previously described for 12 days, with application of the extracts and controls at the end of the 13th day. After 7 days of application, 10 mL of distilled water were applied in each separate slot to leachate the extract away every 2 days until completing 8 days (i.e., 4 washings). The same growth parameters above were measured after the last water application.

#### 5.2.6. Statistical Analyses

Statistical analysis was done with GraphPad Prism 8.1. Results were pre-evaluated for normal distribution by the Shapiro–Wilk or Levene test and then analyzed by one-way ANOVA followed by Tukey test (normal distribution) or Kruskal–Wallis followed by Dunn’s test (non-parametric distribution), all at *p* ≤ 0.05. For the two treatment comparisons, a *t*-test was applied (after normality verification and at *p* ≤ 0.05). Data were expressed as mean ± standard deviation (SD). Germination data were analyzed using 95% confidence intervals (Wilson score interval) [48].

## Data Availability

The original contributions presented in the study are included in the article/Supplementary Material, and further inquiries can be directed to the corresponding author.

## Supplementary Materials

The following supporting information can be downloaded at: https://www.mdpi.com/article/10.3390/plants12030663/s1, Figure S1: Thin layer chromatography of *Quillaja* preparations. AE—aqueous extract prepared from *Q. lancifolia* leaves; Quil-A—commercial preparation obtained from *Q. saponaria* barks; QB-90—purified from AE after column chromatography. Triterpenoids stained brown with anisaldehide-sulfuric. Figure S2: HPLC chromatogram (214 nm) of QB-90 (4 mg/mL) from the leaf extract of *Quillaja lancifolia*. The main triterpene saponins elute in the range of 15 to 25 min. The known saponin QS-21 elutes between 20.5 and 20.7 min. Figure S3: GC-MS analysis of QB-90 after acid hydrolysis. Breakdown of saponins produced quillaic acid as the main aglycone (quillaic acid chemical structure shown on the right). The presence of quillaic acid was confirmed with an authentic standard. IS—internal standard (cholestane). Figure S4. Post-emergence bioassay with barnyardgrass. Parameters (7 days after treatment): (A) shoot length, (B) root length, (C) chlorophyll concentration, and (D) seedling dry weight. Abbreviations: C- (distilled water), AE 4% (aqueous extract at 4%), AE 10% (aqueous extract at 10%) and C+ (NaCl). Bars represent the mean + SD. Different letters indicate significant difference by Kruskal-Wallis and Dunn’s test (A and D) or one-way ANOVA and Tukey test (B and C) (*p* ≤ 0.05). Figure S5. Post-emergence bioassay with barnyardgrass. Seedlings at the end of 7 days treated with (A) distilled water—C- and (B) aqueous extract at 10%—AE 10%. Bar = 1 cm. Figure S6. Post-emergence bioassay with lettuce carried out in petri dishes. Parameters (7 days after treatment): (A) shoot length, (B) root length, and (C) seedling dry weight. Abbreviations: C- (distilled water), AE 4% (aqueous extract at 4%), AE 10% (aqueous extract at 10%) and C+ (NaCl). Bars represent the mean + SD. Different letters indicate significant difference by Kruskal-Wallis and Dunn’s test (*p* ≤ 0.05). Figure S7. Substrate leaching bioassay with lettuce. Seedlings at the end of 12 days in substrate treated with (A) distilled water—C-, (B) aqueous extract at 4%—AE 4% and (C) aqueous extract at 10%—AE 10%. Bar = 1 cm. Figure S8. Substrate leaching bioassay. Germination rates of lettuce seeds in soil-like substrate with the previous application of the following treatments: distilled water (C-), aqueous extract at 4% (AE 4%) and aqueous extract at 10% (AE 10%), during 12 days of experiment. Bars show 95% confidence intervals (Wilson score interval). Treatments not sharing the same letter at the final sampling time differ in germinability (*p* ≤ 0.05). Reference [46] is cited in the supplementary materials.
