# Supplementary material for: Phytotoxicity of Quillaja lancifolia Leaf Saponins and Their Bioherbicide Potential
Source: Plants (Basel). 2023 Feb 2;12(3):663. doi: 10.3390/plants12030663 (PMC9919483; doi:10.3390/plants12030663)
Supplement: Supplementary file 1 [file plants-12-00663-s001.zip › plants-2132288-Supplementary.pdf]

Table S1. Density measured with a refractometer and the respective osmotic potential values of different concentrations of *Quillaja lancifolia* aqueous extract (at 1%, 2%, 4%, 10%, 20% and 40%) and of Polyethylene glycol (PEG-6000) (at 0.01, 0.02, and 0.03 M).

	Density (g/mL)	Osmotic potential (MPa)
AE 1%	1.000	-0.191
AE 2%	1.000	-0.191
AE 4%	1.001	-0.192
AE 10%	1.034	-0.200
AE 20%	1.065	-0.205
AE 40%	>1.120	>-0.215
PEG 0.01 M	1.001	-0.192
PEG 0.02 M	1.042	-0.200
PEG 0.03 M	>1.120	>-0.215

Table S2. Germination Velocity Index (GVI) for lettuce (4 days) and barnyardgrass (5 days). Seeds were treated with the following treatments: aqueous extracts of *Q. lancifolia* at 4% and 10% (AE 4% and AE 10%, respectively), distilled water (negative control, C-), and NaCl 0.5 M (positive control, C+). Different letters within each column indicate significant difference (Wilson score intervals, p≤0.05).

	GVI	
	Lettuce	Barnyardgrass
C-	34.25 a	16.9 a
AE 4%	0 b	2.65 b
AE 10%	0 b	0 c
C+	0 b	0.33 c

Table S3. Viability loss of seedlings at the end of the post-emergence bioassay (7 days after application of treatments). Abbreviations: C- (distilled water), AE 4% (aqueous extract at 4%), AE 10% (aqueous extract at 10%) and C+ (NaCl). Different letters indicate significant difference (Wilson score intervals, p≤0.05).

Treatments	% Dead seedlings (confidence intervals)	Treatment groups
C-	0 (0-4)	b
AE 4%	0 (0-4)	b
AE 10%	24 (20-30)	a
C+	16 (10-20)	a

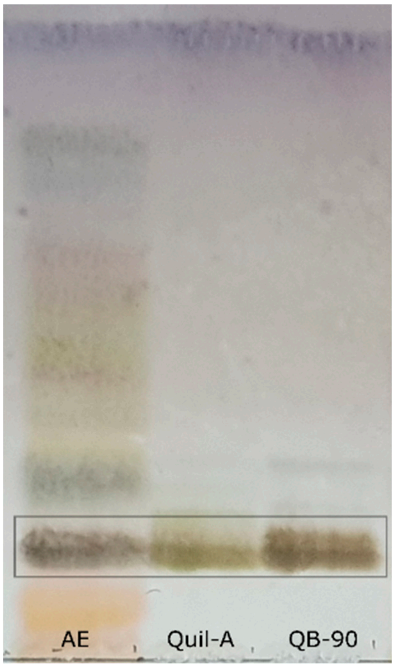

Figure S1. Thin layer chromatography of *Quillaja* preparations. AE – aqueous extract prepared from *Q. lancifolia* leaves; Quil-A – commercial preparation obtained from *Q. saponaria* barks; QB-90 – purified from AE after column chromatography. Triterpenoids (boxed spots) stain brown with anisaldehide-sulfuric.

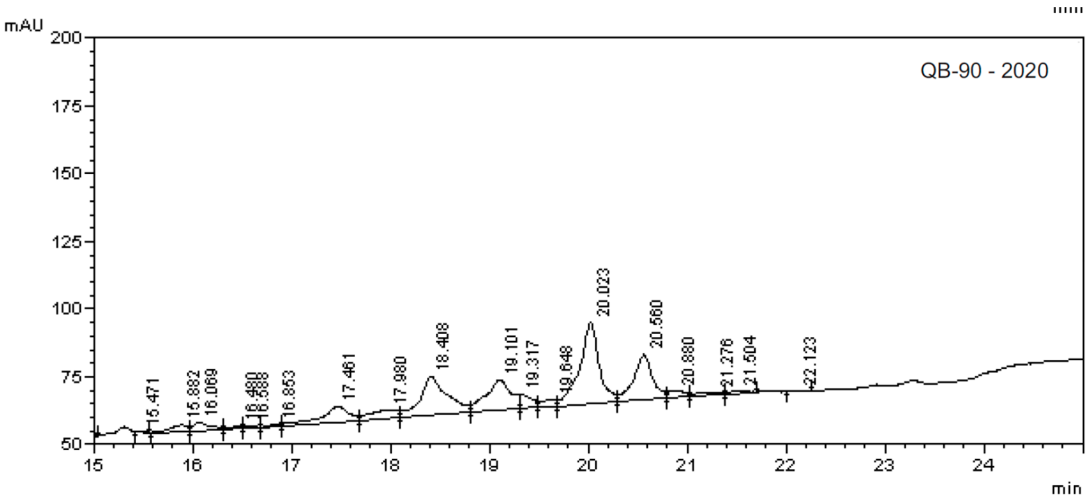

Figure S2. HPLC chromatogram (214 nm) of QB-90 (4mg/mL) from leaf extract of *Quillaja lancifolia*. Main triterpene saponins elute in the range of 15 to 25 min. The known saponin QS-21 elutes between 20.5 and 20.7 min.

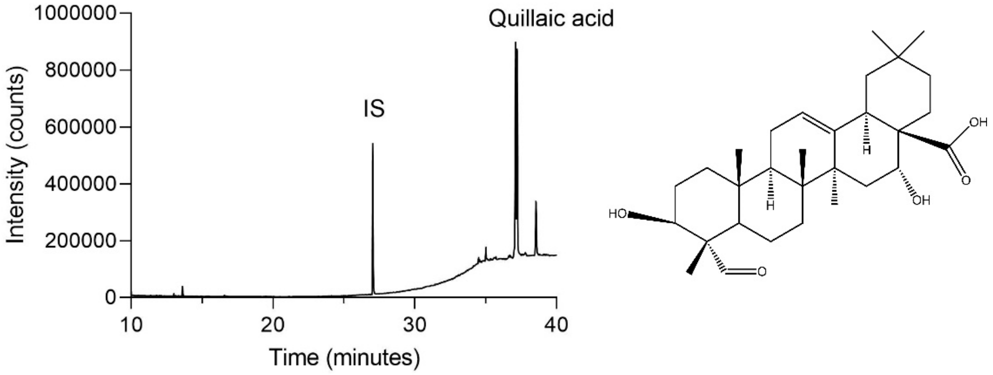

Figure S3. GC-MS analysis of QB-90 after acid hydrolysis. Breakdown of saponins produced quillaic acid as the main aglycone (quillaic acid chemical structure shown on the right). The presence of quillaic acid was confirmed with authentic standard. IS – internal standard (cholestane) [46].

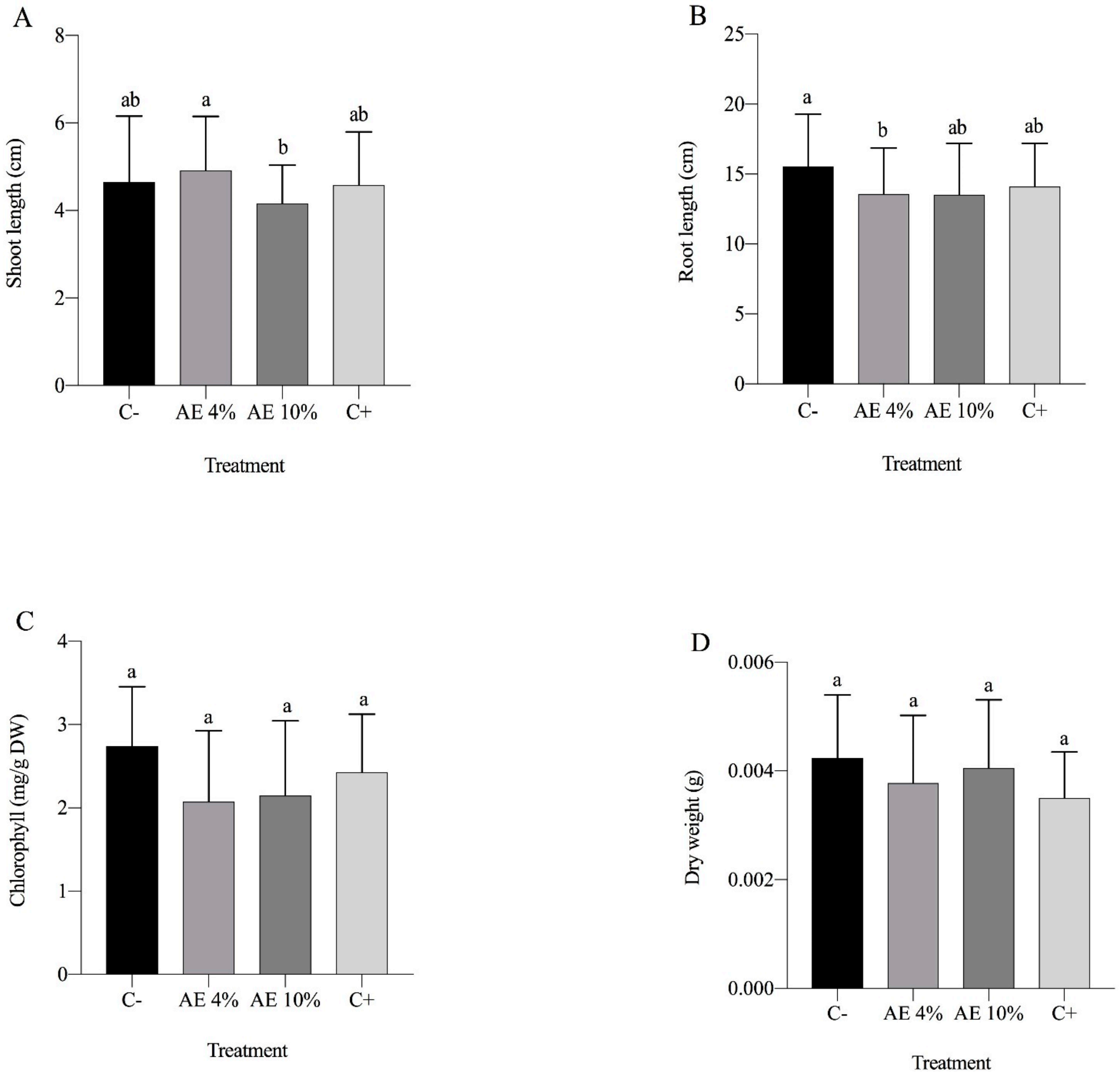

Figure S4. Post-emergence bioassay with barnyardgrass. Parameters (7 days after treatment): (A) shoot length, (B) root length, (C) chlorophyll concentration, and (D) seedling dry weight. Abbreviations: C- (distilled water), AE 4% (aqueous extract at 4%), AE 10% (aqueous extract at 10%) and C+ (NaCl). Bars represent the mean+SD. Different letters indicate significant difference by Kruskal-Wallis and Dunn's test (A and D) or one-way ANOVA and Tukey test (B and C) ($p \leq 0.05$).

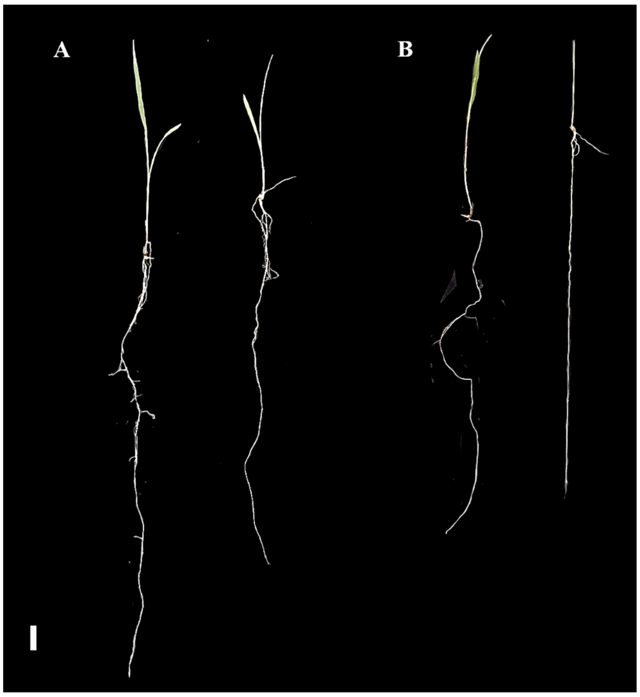

Figure S5. Post-emergence bioassay with barnyardgrass. Seedlings at the end of 7 days treated with (A) distilled water – C- and (B) aqueous extract at 10% – AE 10%. Bar=1cm.

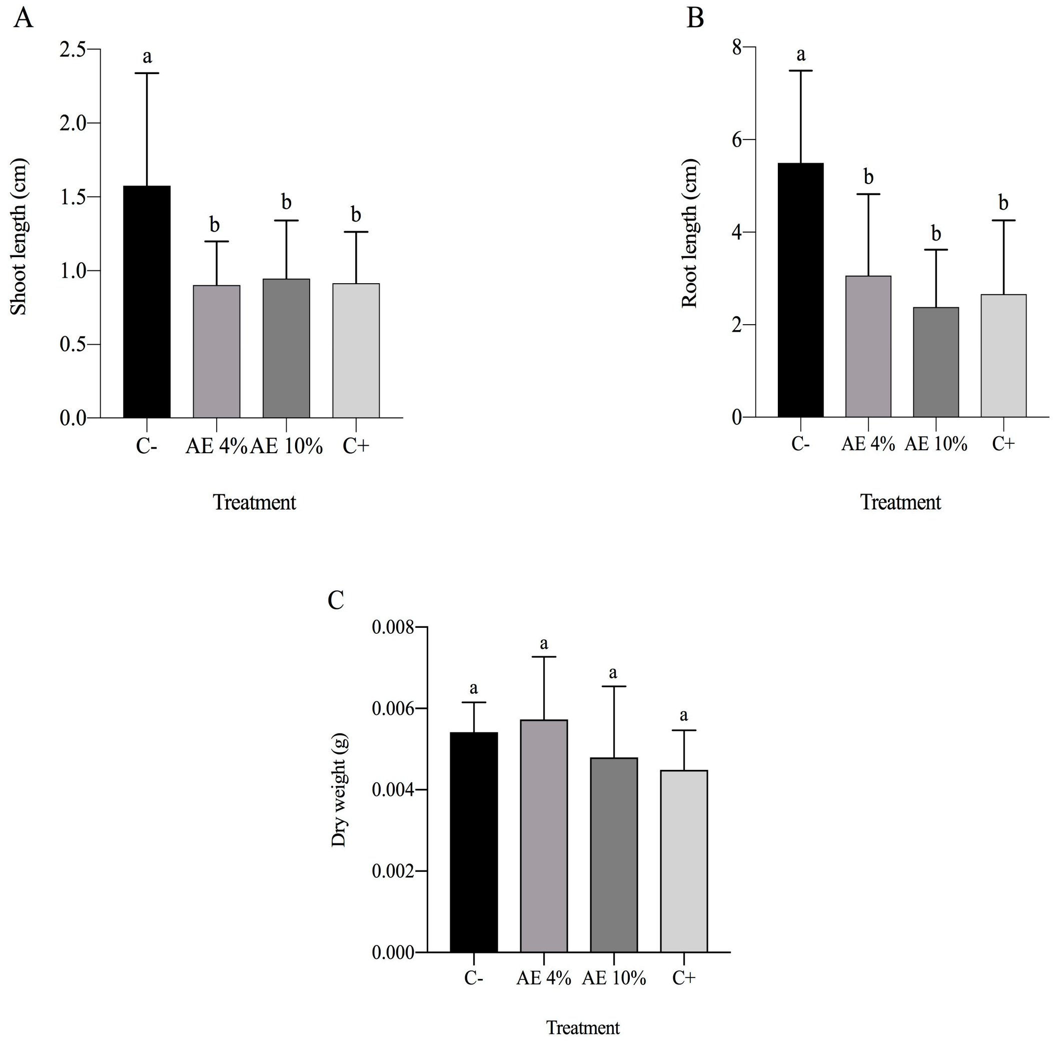

Figure S6. Post-emergence bioassay with lettuce carried out in petri dishes. Parameters (7 days after treatment): (A) shoot length, (B) root length, and (C) seedling dry weight. Abbreviations: C- (distilled water), AE 4% (aqueous extract at 4%), AE 10% (aqueous extract at 10%) and C+ (NaCl). Bars represent the mean+SD. Different letters indicate significant difference by Kruskal-Wallis and Dunn's test ($p \leq 0.05$).

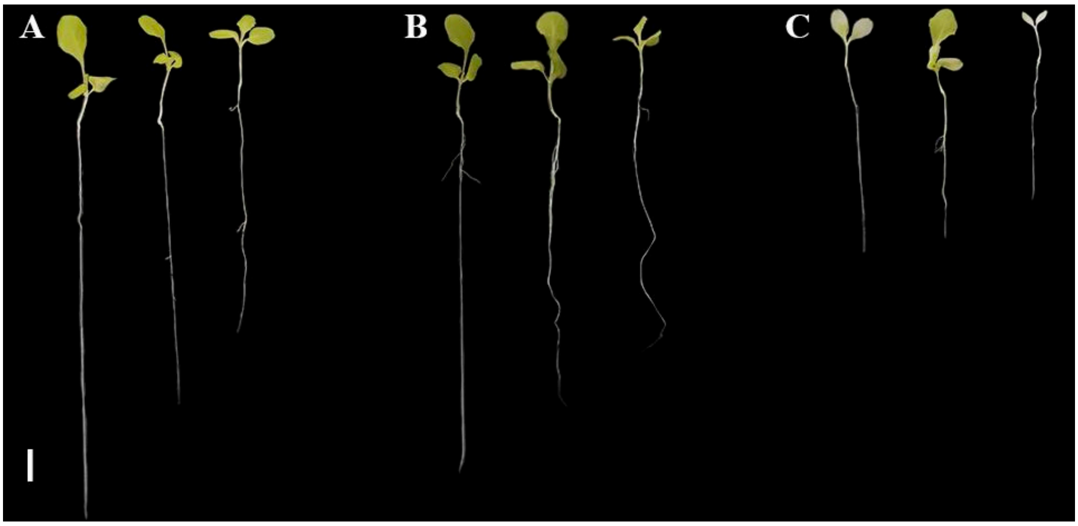

Figure S7. Substrate leaching bioassay with lettuce. Seedlings at the end of 12 days in substrate treated with (A) distilled water - C-, (B) aqueous extract at 4% - AE 4% and (C) aqueous extract at 10% - AE 10%. Bar=1cm.

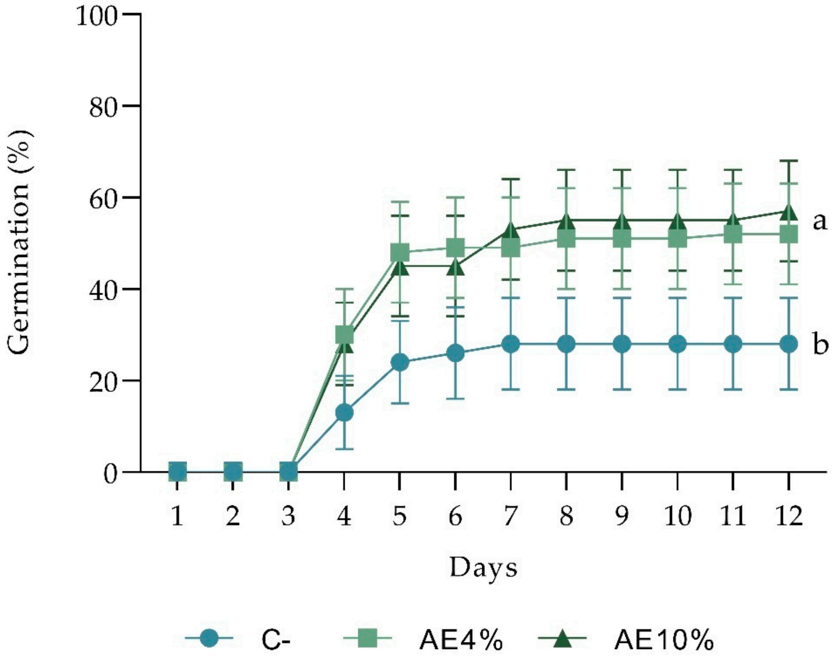

Figure S8. Substrate leaching bioassay. Germination rates of lettuce seeds in soil-like substrate with the previous application of the following treatments: distilled water (C-), aqueous extract at 4% (AE 4%) and aqueous extract at 10% (AE 10%), during 12 days of experiment. Bars show 95% confidence intervals (Wilson score interval). Treatments not sharing the same letter at the final sampling time differ in germinability ($p \leq 0.05$).
